# A Trematode Parasite Derived Growth Factor Binds and Exerts Influences on Host Immune Functions via Host Cytokine Receptor Complexes

**DOI:** 10.1371/journal.ppat.1005991

**Published:** 2016-11-02

**Authors:** Azad A. Sulaiman, Katarzyna Zolnierczyk, Ornampai Japa, Jonathan P. Owen, Ben C. Maddison, Richard D. Emes, Jane E. Hodgkinson, Kevin C. Gough, Robin J. Flynn

**Affiliations:** 1 School of Veterinary Medicine and Science, the University of Nottingham, Sutton Bonington Campus, Nottingham, United Kingdom; 2 School of Medicine, University of Phayao, Phayao, Thailand; 3 ADAS UK, the University of Nottingham, Sutton Bonington Campus, Nottingham, United Kingdom; 4 Department of Infection Biology, Institute of Infection and Global Health, University of Liverpool, Liverpool, United Kingdom; University of Medicine & Dentistry New Jersey, UNITED STATES

## Abstract

The trematode *Fasciola hepatica* is responsible for chronic zoonotic infection globally. Despite causing a potent T-helper 2 response, it is believed that potent immunomodulation is responsible for rendering this host reactive non-protective host response thereby allowing the parasite to remain long-lived. We have previously identified a growth factor, FhTLM, belonging to the TGF superfamily can have developmental effects on the parasite. Herein we demonstrate that FhTLM can exert influence over host immune functions in a host receptor specific fashion. FhTLM can bind to receptor members of the Transforming Growth Factor (TGF) superfamily, with a greater affinity for TGF-β RII. Upon ligation FhTLM initiates the Smad2/3 pathway resulting in phenotypic changes in both fibroblasts and macrophages. The formation of fibroblast CFUs is reduced when cells are cultured with FhTLM, as a result of TGF-β RI kinase activity. In parallel the wound closure response of fibroblasts is also delayed in the presence of FhTLM. When stimulated with FhTLM blood monocyte derived macrophages adopt an alternative or regulatory phenotype. They express high levels interleukin (IL)-10 and arginase-1 while displaying low levels of IL-12 and nitric oxide. Moreover they also undergo significant upregulation of the inhibitory receptor PD-L1 and the mannose receptor. Use of RNAi demonstrates that this effect is dependent on TGF-β RII and mRNA knock-down leads to a loss of IL-10 and PD-L1. Finally, we demonstrate that FhTLM aids newly excysted juveniles (NEJs) in their evasion of antibody-dependent cell cytotoxicity (ADCC) by reducing the NO response of macrophages—again dependent on TGF-β RI kinase. FhTLM displays restricted expression to the *F*. *hepatica* gut resident NEJ stages. The altered fibroblast responses would suggest a role for dampened tissue repair responses in facilitating parasite migration. Furthermore, the adoption of a regulatory macrophage phenotype would allow for a reduced effector response targeting juvenile parasites which we demonstrate extends to an abrogation of the ADCC response. Thus suggesting that FhTLM is a stage specific evasion molecule that utilises host cytokine receptors. These findings are the first to clearly demonstrate the interaction of a helminth cytokine with a host receptor complex resulting in immune modifications that facilitate the non-protective chronic immune response which is characteristic of *F*. *hepatica* infection.

## Author Summary

Parasitic worms, helminths, can cause long-lived chronic infection in many hosts that they infection. The liver fluke, *Fasciola hepatica*, is one such parasite causing global infection of both humans and animals. F. hepatica exerts an influence over the immune system such that it avoids effector mechanisms and prevents the development of effective immunity. Here we characterise a molecule—FhTLM—derived from juvenile parasites that is similar to the regulatory cytokine TGF-β. We show that FhTLM will bind to host TGF-β receptors with a reduced affinity when compared with mammalian TGF-β. Despite this FhTLM can induce Smad2/3 signalling in host leukocytes, which is key to initiating gene transcription. Phenotypically FhTLM causes fibroblasts to slow their growth and replication response resulting in slower wound healing. Importantly FhTLM induces a macrophage phenotype that resembles a regulatory macrophage phenotype identified in other species undergoing helminth infection. Finally we Our work highlights the potential of FhTLM to play important roles in controlling host immunity when initially infected with juvenile parasites, thereby preventing the development of effective immunity.

## Introduction

The trematode *Fasciola hepatica* is capable of establishing chronic infection in multiple hosts that can last for many years. In terms of animal infections *F*. *hepatica* is highly prevalent within sheep, cattle and goats throughout temperate regions of the globe with varying levels of infection reported from 30%-70%[1]. This problem is compounded by a growing degree of resistance against what has been the drug of choice for combating infection, triclabendazole [2]. *F*. *hepatica* however is not solely an animal problem as it also has growing implications for human health with large endemic foci of infection within South America and the Middle East [3]. Crucially, there has now been a reported case of triclabendazole resistant parasites causing human infection [4]. As such *F*. *hepatica* has been added to the list of emerging zoonotic diseases [5]. In response to infection an extremely polarised T-helper (Th) 2 response characterised by high levels of IgG1, IL-4 and eosinophilia. Despite the magnitude of this response naturally infected animals fail to develop immunity [6] and efforts at experimental vaccination have thus far demonstrated that a mixed Th2/Th1 profile is required to achieve a reduction in parasite burdens, egg outputs and liver damage [7]. As infection progresses the Th2 response switches to a characteristic regulatory response—this is denoted by high levels IL-10 and TGF-β and suppression of antigen specific T-cells [8].

Much research has identified the nature and mode of action of parasite derived immunomodulators. Chief amongst these is the aforementioned cathepsin L1 which has also been shown to suppress Th1 cytokine secretion. Cathepsin L1 has also been shown to cleave CD4 from the surface of lymphocytes [9] and prevent antibody-dependent cell cytotoxicity (ADCC) from killing newly excysted juvenile (NEJ) parasites [10]; one of the few mechanisms known to kill NEJs. More recently Donnelly and colleagues have shown multiple modes of immunosuppression involving a family of helminth defence molecules, similar to host defence peptides that are capable of suppressing macrophage activation and B-cell cytokine responses [11]; providing evidence parasite for mimicry.

Another family of proteins which has demonstrated conservation between host and multiple parasites is the Transforming Growth Factor (TGF) superfamily [12]. Members of this protein superfamily have been shown to predominantly play roles in body patterning, optimal sexual development and reproductive success. Members of this family have been found in free-living and parasitic worms including *Schistosoma mansoni* [13], *S*. *japonicium* [14], *Ancylostoma caninum* [15] *and Caenorhabditis elegans* [16]. We have recently demonstrated that the *F*. *hepatica* contains a TGF-like molecule which we termed FhTLM [17]. Previously we have shown that the expression of FhTLM was restricted to the NEJ stage. Further to this we have provided evidence that recombinant FhTLM could enhance motility and survival of NEJs while increasing the rate at which eggs underwent embryonation. Members of the TGF superfamily have been ascribed many roles including within leukocytes. TGF-β is a requirement for the development of both Th17 and Treg cell subsets with the levels IL-6 playing a crucial role in dictating the fate of these cells. TGF-β secreted from Treg cells or other sources can have an anti-proliferative effect on T-cells after TCR stimulation. Macrophage responses to TGF-β are broadly known to result in anti-tumouricidal [18] and anti-inflammatory [19] macrophages. Recently, the effect of TGF-β has been shown to direct the effects of myeloid suppressor cells in *Nippostrongylus brasiliensis* infection thus controlling Th2 immunity within the lung [20]. TGF-β is also known to be crucial to development of fibrosis, this response can be serve to promote pathology via hypertension during *S*. *mansoni* infection [21]. Studies on *Heligmosomoides polygyrus* suggests that there is a protein(s) which can bind the mammalian TGF-receptor complex and initiate a Smad2/3 signalling program [22]. This *H. polygyrus* derived antigen was found to upregulate FoxP3 expression within naïve T-cells, directly generating Tregs; a finding that explains the protective effect of *H*. *polygyrus* in lung inflammation [23].

Given the above and our own findings with regard to FhTLM we sought to determine if FhTLM could directly interact with the native receptor complex and initiate a phenotype. To begin this process we determined if FhTLM could generate a response signal using a luciferase reporter cell line and physically bind host TGF-β RI and RII. FhTLM initiates a Smad3 signal and can alter the responses of fibroblasts in a TGF-β RI kinase dependent fashion. Moreover when used to activate macrophages the response to FhTLM and the resultant phenotype resembled a regulatory macrophage rather than the helminth-linked alternatively activated macrophage; with high levels of IL-10 and PD-L1 and moderate arginase-1 activity. These processes occurred in a *tgf-βRII* dependent fashion as demonstrated by siRNA knockdown. Finally, the FhTLM macrophage phenotype was incapable of killing NEJ parasites by ADCC demonstrating that this stage specific parasite protein might elicit non-protective responses from resident cells within the intestinal phase of infection.

## Results

### *F*. *hepatica* expresses a growth factor similar to TGF-β

**Fig 1:**
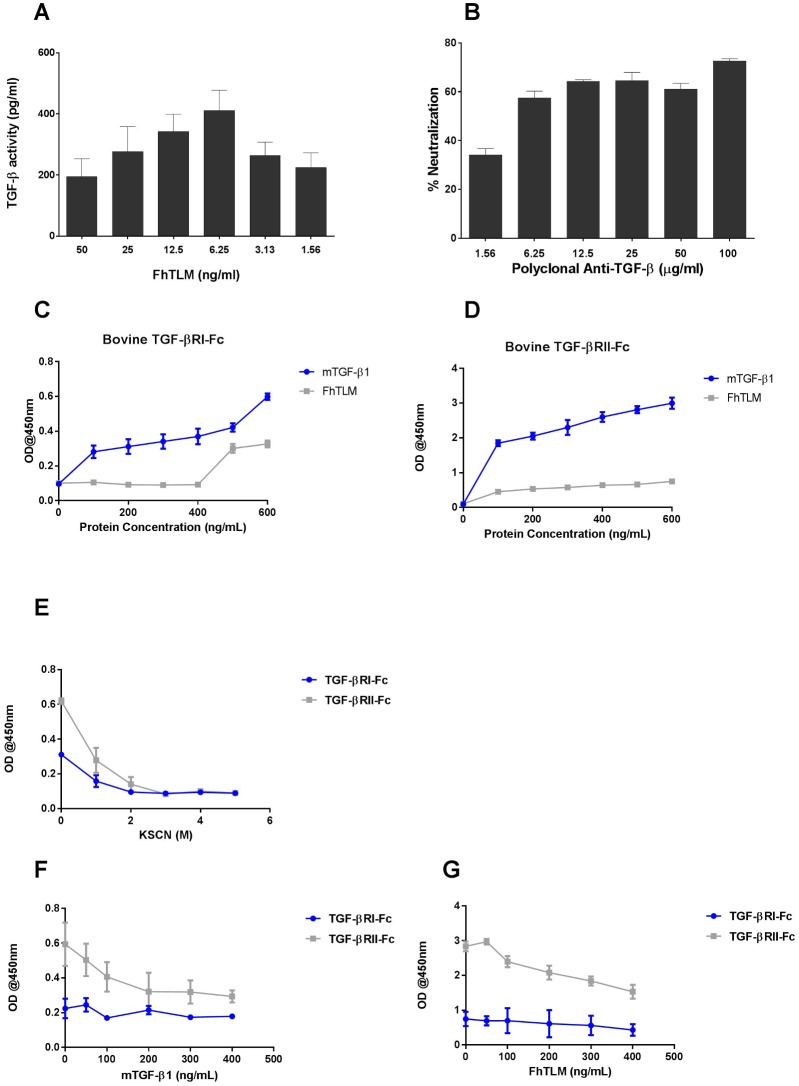
FhTLM binds to and initiates mammalian host signalling pathways. (A) FhTLM was used to stimulate the TGF-β responsive cell line at the indicated doses. Relative light units (RLU) were converted to equivalent TGF-B concentration by comparison with a TGF-β standard curve. (B) This activity was inhibited by polyclonal serum raised against TGF-β. Plate-bound FhTLM was detected using the recombinant fusion proteins (C) bovine TGF-B R1-Fc and (D) bovine TGF-B RII-Fc. Results are reported as optical densities (OD) at 450nm. (E) Avidity of receptor-FhTLM interactions were measured using KSCN and presented as OD as a function of increasing KSCN molarity. (F) and (G) FhTLM and TGF-β, respectively, were used to coat ELISA plates (400ng/mL) overnight. Thereafter TGF-β1 and FhTLM were used in increasing concentrations shown to block the interaction of TGF-βRI-Fc or TGF-βRII-Fc. Data presented are means +/- SD of triplicates, experiments were repeated three times with similar results.

We have recently shown that the trematode parasite *Fasciola hepatica* contains three ligand members of the transforming growth factor superfamily [17]. These include two bone morphogenic proteins (BMPs) and an activin-like molecule which we have terms *F*. *hepatica* TGF-like molecule, FhTLM. We have demonstrated a restricted pattern of expression within the parasite with the highest level of expression within the newly excysted juvenile that emerges within the intestine of hosts. To determine if FhTLM is a bioactive molecule similar to TGF-β we assessed its activity in a reporter assay [24]. A dose response analysis suggests that FhTLM can indeed generate a positive luciferase signal and when compared to a TGF-β1 standard curve it would suggest that FhTLM has a lower degree of bioactivity in this assay when compared with mammalian equivalents (Fig 1A). We were also able to demonstrate a similar activity within crude parasite homogenate (LFH) which required higher concentrations to induce comparable responses (S1 Fig). Initial attempts to use a monoclonal antibody to inhibit FhTLM activity did not demonstrate inhibitory capacity. However, a polyclonal anti-sera raised with broad specificity was found to reduce the activity of FhTLM in the same bioassay in a dose dependent manner (Fig 1B). To ensure the effects of FhTLM were dependent on ligand-receptor based interactions we sought to determine if FhTLM could bind either of the bovine TGF-β RI or RII. We cloned and expressed fusion proteins comprised of the bovine TGF-β RI and RII extracellular domain fused to the human IgG1 Fc domain (S1 Table). Using these proteins we performed a comparison between the binding of these fusion proteins to FhTLM and human TGF-β1 (Fig 1C & 1D –note differences in Y-axis scale). Using both fusion proteins we could confirm that human TGF-β1 could bind the bovine receptors RI and RII. More interestingly we also confirmed that FhTLM could cause the binding of both fusion proteins with a greater affinity for TGF-β RII-Fc, which is similar to the reported affinity of TGF-β RII with TGF-β1 elsewhere [25, 26]. Final confirmation of the greater affinity of TGF-β RII with FhTLM was confirmed by repeating the above assays but with the inclusion of increasing concentrations of KSCN after initial addition of fusion proteins to the plate. A greater concentration of KSCN was required to disassociate the interaction between FhTLM with TGF-β RII-Fc when compared with TGF-β RI-Fc (Fig 1E). Moreover when we tested the affinity of FhTLM for either TGF-β RII-Fc or TGF-β RI-Fc in a competition assay we were able to demonstrate that FhTLM was only moderately able to reduce the binding of free TGF-β RII-Fc or TGF-β RI-Fc to immobilised TGF-β1 reducing binding to TGF-β RII-Fc and TGF-β RI-Fc by 46% and 42%, respectively. In comparison TGF-β was able to reduce binding of FhTLM to TGF-β RII-Fc and TGF-β RII-Fc by 50% and 22%, respectively, as comparable doses (Fig 1F & 1G).

### FhTLM drives Smad2/3 signalling with delayed kinetics

**Fig 2:**
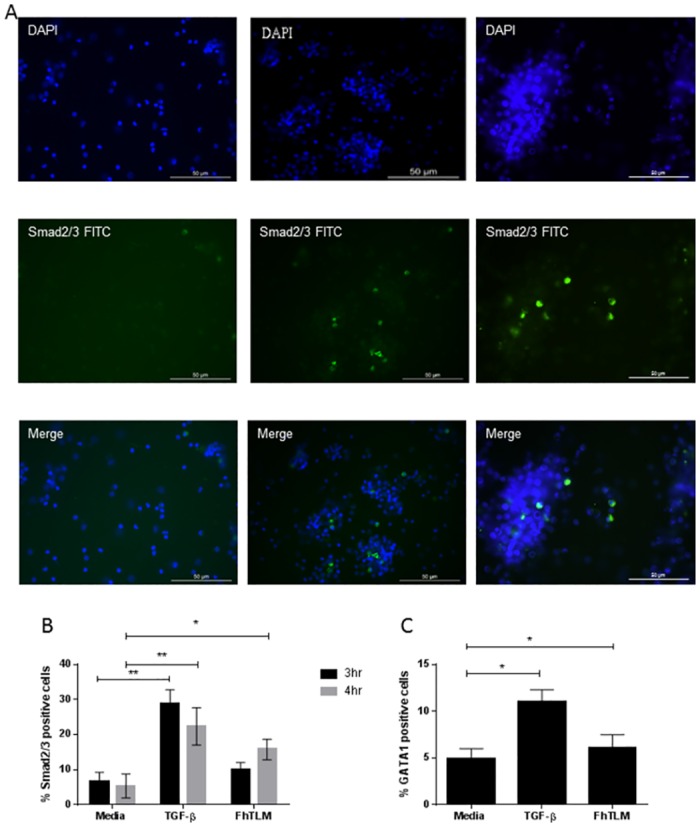
FhTLM can initiate transcription factor activation in primary host leukocytes. Naïve bovine PBMCs were incubated with FhTLM (200ng/mL) and subsequently prepared for immunoflouresence (A) to detect (B) Smad2/3 and (C) GATA1. (A) The upper row are cells stained with DAPI, middle row with anti-Smad2/3-FITC, and bottom row iIn both panels, the columns represent (from left to right) unstimulated PBMCs, TGF-β1 stimulated PBMCs, and FhTLM stimulated PBMCs. Following staining cytospins were examined at 40X magnification and the number of positive cells (i.e. FITC overlaid on DAPI) were calculated. This analysis was conducted over time for Smad2/3 (B) and at 3hrs for GATA1 (C). Data presented are means +/- SEM of triplicates, experiments were repeated three times with similar results. Results were analysed using a 1-way Anova and statistical significant differences are indicated on the graphs, *P<0.05 or **P<0.01.

The TGF-β receptors are G-protein coupled receptors and after ligand binding heterodimers of TGF-β RI and RII are formed. The resultant phosphorylation of this receptor complex triggers movement of the signalling proteins phosphorylated (p)Smad2/3 into the nucleus where gene transcription is initiated [27]. To determine if FhTLM was capable of driving Smad2/3 signalling after engagement with the receptor complex we used primary bovine peripheral blood mononuclear cells (PBMCs) in a stimulation assay to measure the extent of co-localisation of pSmad2/3 with the nucleus. Given the differences we noted above between activity of recombinant FhTLM in our luciferase assay and the binding data determined from our receptor fusion protein assays we conducted a dose response curve for bovine macrophages using IL-10 as our readout to determine the optimal working concentration (S2 Fig). PBMCs were stimulated for between 3 and 4hrs, fixed and stained with DAPI (Fig 2A top row), anti-pSmad2/3-FITC (Fig 2A middle row) and the percentage of co-localisation (or pSmad2/3 positive cells) was determined (Fig 2A bottom row). TGFβ clearly drives pSmad2/3 signalling in this setting with 28% (±4.1%) of cells being pSmad2/3 positive at 3hrs post-stimulation with a small, but not significant decrease, at 4hrs to 22.4% (±5.3%). Interestingly when we initially examined cells stimulated for 3hrs with FhTLM we found only 10.7% (±1.9%) of cells positive which was not significantly increased when compared to our controls [6.8%(±2.5%)]. However, when we extended our analysis to 4hrs we found that FhTLM induced pSmad2/3 in 15.8% (±2.9%) of cells which was significantly different when compared to controls [5.4% (±3.4%)]. A recent analysis of the bovine *il10* promoter within our lab has indicated a role for GATA1 in driving *il10* expression. Furthermore GATA1 has been in implicated in anti-helminth immunity in previous studies using *Nippostrongylus brasiliensis* [28] and *S*. *mansoni* [29]. We subjected PBMCs to a 4hr stimulation as above and then determined the levels of GATA1 co-localisation and found that both TGF-β (11%±1.3%) and FhTLM (6.1%±1.4) could induce significantly more GATA1 co-localisation in PBMCs when compared to controls (4.9%±1.1). Our results clearly demonstrate that FhTLM can act to induce both direct and indirect transcription factors in primary host PBMCs.

### FhTLM alters fibroblast growth dynamics

**Fig 3:**
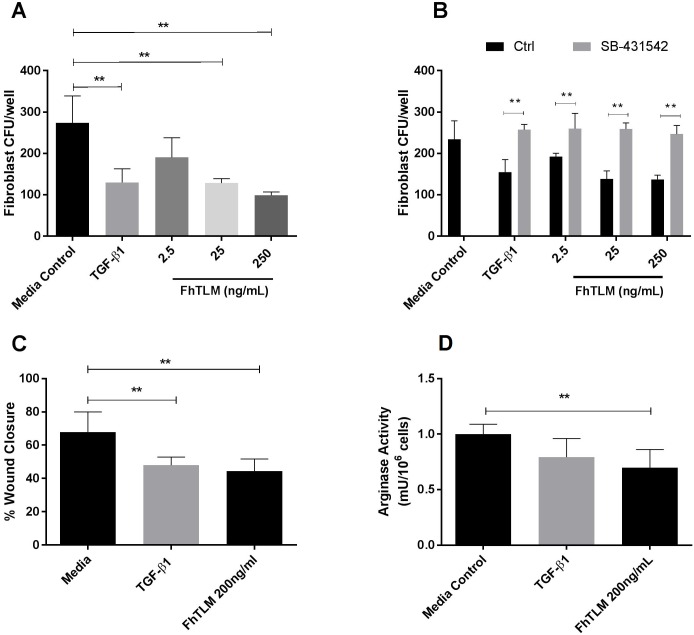
FhTLM changes fibroblast responses. FhTLM was included as an additive in fibroblast CFU cultures (A) or in wound repair assays (C). (A) 3T3 fibroblasts were seeded at 6 cells/well in 6 well plates with the indicated additives. Cultures were incubated for 10 days, stained with crystal violet and CFUs were counted. (B) Cultures as above were established but with the addition of the specific TGF-β RI activin receptor-like kinase (ALK) inhibitor SB431542 (5μM). (C) 3T3 fibroblasts were seeded in 6 wells plates and grown to 80% confluence. Horizontal wounds were introduced and wells imaged at T0 and again at T24. TGF-β or FhTLM were included post-wounding at the indicated concentrations. Images were blinded and % wound closure was calculated as area of well visible at T24 relative to T0. (D) Cell lysates were collected from (C) and arginase levels were determined by enzyme assay and reported as mU/10^6^ cells. For A, B and C data represents means +/- SEM of 4–6 measurements and experiments twice were repeated with similar results. Data in D represent combined data from (C) with N = 8–12 measurements +/- SEM per treatment. Results were analysed using a 1-way Anova and statistical significant differences are indicated on the graphs, P **P<0.01.

Characterisation of TGFβ demonstrated a profound an anti-proliferative and developmental effect on multiple cell types [30–35]. In an effort to ascribe a phenotype to the effects of FhTLM we performed CFU assays using the NIH 3T3 fibroblast line. Cells were seeded at a density of 6 cells/petri dish and incubated for 10 days. This was done in the presence of TGF-β or increasing concentrations of FhTLM (2.5, 25, 200 ng/mL). CFUs that formed were counted and our results clearly show a significant decrease in number of CFUs that formed when higher doses of FhTLM were used (Fig 3A). 25ng/mL of FhTLM was sufficient to reduce the number of CFUs to comparable level as seen on those incubated with TGF-β (128.3±6.173 vs. 129.7 ±19.10). To further determine the effects of FhTLM on fibroblast activity we performed *in vitro* scratch assay/wound closure experiments. Confluent cells were scratched and imaged before incubation for 24hrs with TGF-β or FhTLM. After 24hrs the wounds were imaged and total area determined (Fig 3B). Expressing this area as % wound closure we found that both TGF-β and FhTLM significantly reduced wound closure (P<0.01) [Ctrl = 67.77%±12.17 vs. TGF = 47.95±4.906 vs. FhTLM = 44.47±7.235]. Decreased arginase-1 has been previously shown to correlate with delayed wound resolution [36]. We determined the levels of arginase-1 in wounded cultures 24hr after incubation (Fig 3C). We confirmed that in cultures treated with TGFβ or FhTLM the levels of arginase-1 were decreased suggesting an ability of FhTLM in altering arginase-1 activity. We next using chemical inhibition to block the kinase activity of TGF-β RI to determine if the effects of FhTLM are indeed TGF-β dependent and specific. As can be seen in Fig 3B when cells were co-cultured with the inhibitor SB- 431542 [37] during formation of fibroblast CFUs the effect of both TGF-β and FhTLM were abolished. These results suggest that FhTLM can both bind and initiate a specific signal via the TGF-β receptor complex.

### FhTLM induces a regulatory macrophage phenotype dependent on TGF-β RII

**Fig 4:**
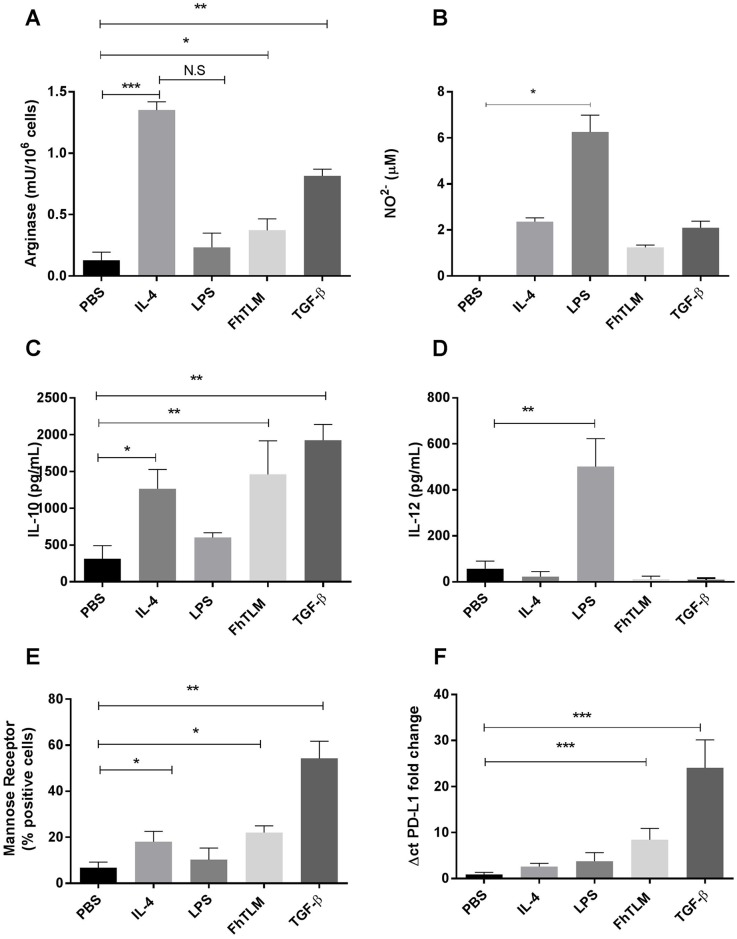
FhTLM induces a part alternative part regulatory macrophage phenotype. FhTLM (200ng/mL) was used to stimulate the bovine macrophages derived from blood monocytes. Thereafter levels of (A) nitric oxide (NO) levels, (B) arginase-1 levels, (C) IL-10, and (D) IL-12 in matched samples. Data in A-D is collected from the same experiment. In separate experiments using the same donors mannose receptor expression was also determined using immunofluorescence (E) and (F) increases in PD-L1 mRNA expression levels were quantified. Data represents means +/- SEM of triplicate measurments and experiments were repeated three times with similar results. Results were analysed using a 1-way Anova and statistical significant differences are indicated on the graphs; *P<0.05, **P<0.01.

Having demonstrated a role for FhTLM in modulating cell growth and regulating the arginase levels of these cells we sought to determine if the effects of FhTLM on arginase levels could be extended to macrophages. The classical and alternative pathways for macrophage activity can be broadly defined in terms metabolism of L-arginine either using iNOS or arginase following stimulation with LPS/IFN-γ or IL-4, respectively [38]. We produced bovine macrophages using purified blood derived CD14^+^ monocytes; these were then stimulated with IL-4, LPS, TGF-β or FhTLM. Our initial analysis confirmed that IL-4 and LPS act to induce arginase or NO, respectively (Fig 4A & 4B). While FhTLM can induce a slight increase, similar to that seen in response to induced TGF-β, in the levels of arginase-1 this was not significant when compared with IL-4 but was significantly different when compared with the control. Similarly LPS induced NO but IL-4, TGF-β or FhTLM induced marginal levels in comparison. To further determine if FhTLM could alter the phenotype of macrophages we measured IL-10 and IL-12. Only LPS stimulation induced significantly more IL-12 when compared to controls (Fig 4D). However, both IL-4, TGF-β and FhTLM induced IL-10, raising levels significantly above controls (Fig 4C). While TGF-β tended towards higher levels of IL-10 induction when compared with L-4 this was not significant. Reports of a regulatory macrophage phenotype in helminth infection or in response to helminth products suggest that this cell population is distinct from alternatively activated macrophages [39, 40] and in some cases it would appear to be independent of arginase-1 [39]. These reports suggest that upregulation of mannose receptor (MR) and PD-L1 serve as proxy markers for these cells. We determined MR levels in stimulated cells by immunofluorescence or PD-L1 levels of qPCR (Fig 4E & 4F). FhTLM significantly upregulated MR expression compared to controls and the number of cells becoming MR^+^ after stimulation was comparable to IL-4 treatment. However TGF-β, in comparison to both IL-4 and FhTLM, was induced a higher number of MR^+^ cells [~60% vs 20%] (Fig 4E). When we examined PD-L1 expression only FhTLM and TGF-β were able to induced PD-L1 above levels seen in controls, again with TGF-β inducing higher levels of PD-L1 compared to FhTLM [30 fold change vs 10 fold change] (Fig 4F).

**Fig 5:**
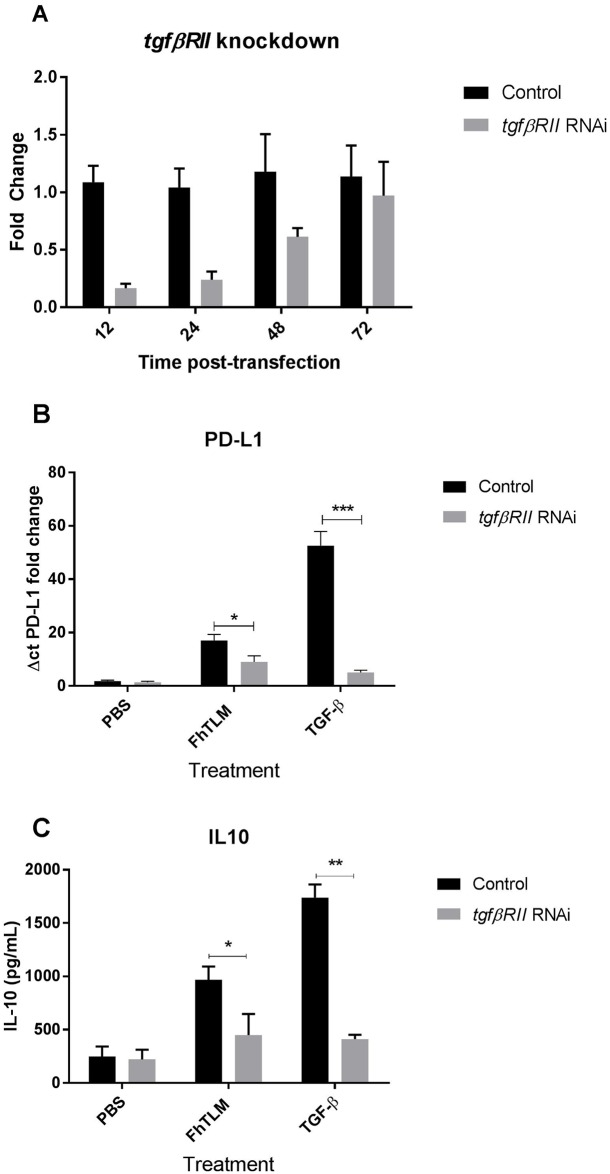
FhTLM requires TGF-β RII to induce IL-10 and PD-L1. To access whether the effects of FhTLM on macrophages was specific to interactions with the host TGF-β receptor complex we employed siRNA to knock-down *tgf-β RII* expression. Primary macrophages were isolated and prior to stimulation were transfected with siRNA against *tgf-β RII* using Lipofectamine. (A) Expression of *tgf-β RII* mRNA were quantified by qPCR at the indicated timepoints post-transfection to monitor the effects of knock-down. At 12hrs post-transfection cells were treated with either FhTLM or TGF-β and TGF-β before IL-10 was measured 48hrs after stimulation (B) or PD-L1 was measured 6hrs after stimulation. All data is mean +/- SEM of triplicate measurements, (A) is data from one donor animal presentative of three individuals and data in (B) and (C) represent the means of 4 individual donor animals. Results in (B) and (C) were analysed using a 1-way Anova and statistical significant differences are indicated on the graphs; *P<0.05, **P<0.01, ***P<0.001.

To determine what host factor confers specificity on interaction of FhTLM with bovine macrophages we employed siRNA directed against the *tgf-βRII*. Primary bovine macrophages were as standard, however at point prior to normal cytokine stimulation cells were transfected with target siRNA or with scrambled siRNA, thereafter we measured changes in *tgf-βRII* levels over a 72hr time period. Our results show we could reliably suppress *tgf-βRII* mRNA levels up to 24hrs post transfection (Fig 5A). We then proceeded to stimulate macrophages 6 hours after knock-down, with FhTLM or TGF-β. Our results show that absence of *tgf-βRII* mRNA at the time of cytokine treatment results in a reduced levels of PD-L1 being upregulated in response to both FhTLM and TGF-β with knock-down reducing PD-L1 upregulation by 47% and 90%, respectively (Fig 5B). While in the case of IL-10 induction, measured 54 hours post-transfection and 48hrs post-stimulation, we same similar reductions in the levels of IL-10 in response to FhTLM and TGF-β (Fig 5C). These results strongly support the conclusion that the effects of FhTLM are dependent on the host cytokine receptor complex TGF-β RI and RII, especially when taken in together with our findings above showing that ALK inhibition also negated the effects of FhTLM.

### FhTLM can negate the effects of ADCC on juvenile *F*. *hepatica*

**Fig 6:**
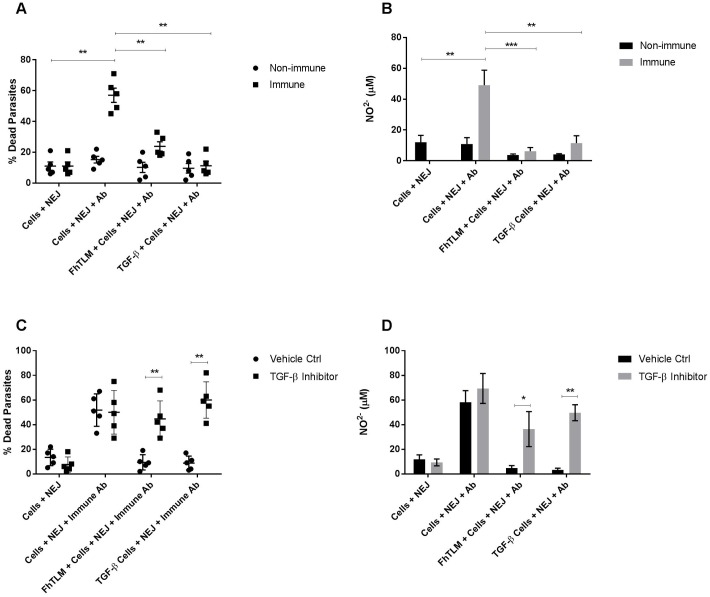
FhTLM reduces the capacity of macrophages to kill juvenile F. hepatica by ADCC. Macrophages were cultured from naïve donors as described in the presence of FhTLM or TGF-β for 24hrs. Following this macrophages were incubated with newly excysted juveniles (NEJs) in the presence of serum taken from naïve (non-immune) or experimentally infected, 13 weeks post-infection, animals (immune). After 6hr of incubation dead parasites were counted (A) and levels of NO in the supernatant determined (B). Experiments were repeated to test the effect of TGF-β RI ALK inhibitor, SB431542 (5μM),on macrophages before incubation with NEJs and serum and measurement of parasite killing (C) and NO levels (D). Data in (A) and (B) were tested for statistical significance using a 2-way anova to determine effect of non-immune vs immune sera and between PBS, FhTLM, and TGF-β macrophage treatment. Data in (C) and (D) were tested using 1-way Anova to determine the effect of inhibitor on TGF-β or FhTLM. **P<0.01 and ***P<0.001. Data displayed is mean +/- SEM and is representative of experiments conducted with 5 individual naïve macrophage donors, using 5 technical replicates per animal.

We have previously shown that FhTLM is selectively expressed within the newly excysted juvenile (NEJs) stage of F. hepatica infection [17]. NEJs are thought to be resident within the intestine for only a number of hours before entering the peritoneal cavity and continuing on their migration to the liver. Within the intestine, multiple type-2 effector responses could be active including antibody-dependent cell cytotoxicity (ADCC). ADCC is one of the few mechanisms shown to actively kill *F*. *hepatica* and has previously been shown to target NEJs, both *in vitro* and *in vivo;* moreover it has previously been shown to be a target of parasite immune evasion mechanisms [10, 41, 42]. We sought to determine if FhTLM altered the macrophage component of this process to benefit parasite survival. Using naïve donor macrophages we incubated cells and NEJs in the presence of either immune or naïve sera. Thereafter viable parasites were counted by visual inspection, as can be seen in Fig 6A the presence of macrophages plus immune sera, but not non-immune sera, resulted in the death of NEJs and was accompanied by induction of NO (Fig 6B). When macrophages were incubated with TGF-β or FhTLM prior to addition of NEJs and sera a different outcome was recorded. As can be seen in Fig 6A both TGF-β and FhTLM reduced the capacity of immune sera to induce ADCC-mediated death of NEJs, this was also accompanied by a loss of NO production (Fig 6B).

We next determined if the effect of FhTLM was dependent on the TGF-β RI, via kinase activity, pre-incubation of cells with the inhibitor SB-431542. Pre-incubation of macrophages with inhibitor prior to TGF-β or FhTLM reversed the negative effect on cells and rescued the ADCC response to NEJs in the presence of immune sera in comparison to cells pre-incubated with vehicle only (Fig 6C). To accompany this we also found that the NO response was restored in both TGF-β and FhTLM cultures in cells pre-incubated with inhibitor but not vehicle only (Fig 6D). Thus our findings demonstrate that FhTLM alters the host macrophage phenotype, via TGF-β RI and RII, to evade ADCC killing of NEJs.

## Discussion

Multiple studies have shown that chronic infection with *F*. *hepatica* can be long lived and accompanied by parasite-specific and non-specific immunosuppression [8, 43, 44]. As the host progresses from a Th2 type response to a more regulatory response it is assumed that secretion of IL-10 and TGF-β increases as a result of either T-cell phenotypic changes or the expansion of T-regulatory cells. The parallel expansion of both Th2 and Treg populations has previously been demonstrated in *S*. *mansoni* [45] and recent work in murine models of *F*. *hepatica* have also demonstrated that infected mice generate a FoxP3^+^ population of cells as infection progresses [46]; however the relevance of this to ruminant immune responses remains to be determined. All of these mechanisms would appear to be driven, or at executed, by the host as a balance to minimise immunopathology. Here we demonstrate that *F*. *hepatica* can utilise a host-exogenous cytokine/growth factor, FhTLM—previously shown to have developmental functions, to direct the host immune system. Indeed this mechanism fits with previous patterns identified whereby for optimal host and parasite survival a balance of immune effector mechanisms must be maintained, allowing parasite survival while avoiding immunopathology [47].

Our data demonstrates that FhTLM is capable of directly engaging TGF-β RI and RII in an ELISA format which makes use of fusion proteins of RI and RII. This data helps to explain the activation by FhTLM of the luciferase reporter. Indeed there are prior reports of parasite derived molecules driving activation in this assay system previously [22]. What is apparent from these data is that FhTLM has a) a higher affinity for TGF-β RII over RI and b) has a lower binding capacity for either receptor when compared with human TGF-β1; this is further evidenced by our competition data. This effect is seen again in the results of our luciferase assay whereby FhTLM was needed in the ng/mL range to generate signals equivalent to those seen in TGF-β in the pg/mL range. The higher affinity of TGF-β for TGF-β RI and RII is also seen in mammalian systems [25, 26] and highlights the conservation between the two ligands despite being phylogenetically distinct, demonstrating the close association amongst the parasite and host. To ensure that the immunomodulatory capacity of FhTLM was not due to binding but not initiating signalling from the receptor complex we examined the canonical intracellular signalling molecule Smad2/3. Using immunofluorescence we can see that not only does FhTLM drive p-Smad2/3 translocation to the nucleus but also does so at slower rate when compared to mammalian TGF-β, again in line with our findings from our binding experiments. It took 4hrs of stimulation with FhTLM to drive a p-Smad2/3 signal distinct from background when compared with the higher translocation rate and shorter time period required in response to TGF-β.

TGF-β is pleiotropic in terms of its effects being implicated in developmental processes, anti-proliferative in a context dependent fashion, responsible for fibrosis, and key to differentiation of two distinct CD4^+^ T-helper subsets. TGF-β is known to be anti-proliferative in terms of fibroblasts [30, 34, 35] and we confirm this finding here and also demonstrate that FhTLM can cause a similar response which is also dependent on TGF-β RI kinase activity. FhTLM reduced the number of CFUs formed to a similar rate of TGF-β over a 10 day period. Likewise when included as a growth factor in *in vitro* wound assays we found that FhTLM, like TGF-β, reduced the rate of wound closure. The role of TGF-β in wound responses is still disputed with some reports finding a positive or negative role dependent on the phase of wound resolution in which it is examined. A recent study however determined that arginase-1 was crucial for healing in murine model wounding [36] and here we found that in parallel with reducing wound closure FhTLM also reduced the cellular levels of arginase-1. Campbell et al [36] found the reduction in arginase-1 levels also resulted in a reduction in pro-inflammatory cell recruitment, including macrophages. The benefits in delayed wound healing during a parasite infection are not apparent however given the migratory nature of *F*. *hepatica* infection, it could be speculated that reducing the rate at which wounds or migratory paths caused by the parasite are healed may confer a benefit to the parasite. The parasite excysts within the intestine and migrates into the peritoneal cavity where it gains access to liver before moving to bile ducts [48]. As the parasites migrate through the intestine they formed a cavity around themselves which would require healing post migration. The delay in healing may increase the rate of successful migrating NEJs; it is already knwon that NEJs secrete proteases to digest surrounding tissue to facilitate their movement [49].

Given the context specific effects of TGF-β we sought to determine its effects on other cell types. Studies suggest that helminth infection [39] and a recombinant helminth immunomodulator [40] can induce a macrophage phenotype that is distinct from the alternatively activated phenotype that is normally associated with helminth infection [50]. Our data demonstrated a subtle yet significant rise in arginase-1 levels following FhTLM, in contrast to our results in the fibroblast experiments, and no increase in NO and in comparison to the strongly polarising effect that IL-4 has on these readouts the results were not striking and more akin to the response to TGF-β. This pattern concurs with the findings of Smith et al.,[39] who found a helminth elicited macrophage population could protect from colitis but in an arginase-1 dependent manner. We next examined the cytokine profile, IL-10 and IL-12, of these cells we found a more pronounced effect of FhTLM. FhTLM could upregulate IL-10 while also suppressing the expression of IL-12, this has been a reported feature of regulatory macrophages for some time [51]; again this pattern of responses being more similar to TGF-β than IL-4. Moreover mRNA expression of PD-L1 and the number of mannose receptor positive cells were significantly upregulated in FhTLM or TGF-β treated macrophages only. During infection with *Taenia crassiceps* PD-L1 has been shown to suppress T-cell responses and neutralisation of PD-L1 on macrophages from infected mice abrogated their suppressive capacity [52]. Recently, an *Ancanthocheilonema viteae* derived immunomodulatory was shown to induce macrophages with high levels of expression of IL-10 and PD-L1 capable of reducing signs of colitis in mice after cell transfer [40]. The mannose receptor (CD206) has previously been shown to be upregulated on regulatory macrophages from a variety of settings [53, 54] including controlling their role in regulating inflammatory cytokine release in Pneumocystis infection [55] and endotoxin-induced lung injury [56]. We found the effects of FhTLM on PD-L1 and IL-10 to be dependent on tgf-βRII expression, as use of siRNA resulted in a loss of their expression following stimulation. Given the effects of FhTLM on macrophages and the restricted expression of FhTLM to NEJs within the parasite itself; we sought to determine the effects of FhTLM on a NEJ targeting effector mechanism—ADCC. ADCC has been shown to kill NEJs when using cells from cattle [41], rats [42], and mice [10] but not sheep [57].This is thought to be as a result of a lack of NO generation in sheep macrophages. Here we demonstrate that bovine macrophages, in the presence of immune sera, kill NEJs and release NO into the supernatant. Moreover the culture of macrophages with FhTLM or TGF-β prior to this assay effectively removed the killing phenotype and reduced the levels of NO. When these assays were repeated in the presence of the TGF-β RI kinase inhibitor the ADCC effect was rescued and parasites were rendered non-viable and NO levels were restored, again showing the effects of FhTLM to be TGF-β receptor dependent.

The expression of TGF-β homologues within helminth parasites has been previously identified [58] however this, to our knowledge, is the first full description of the suppressive effect of a recombinant helminth TGF-β homologue on its host immune system. Our findings indicate a role for FhTLM in the modulation of host macrophages to avoid a well-recognised mechanism of killing ADCC. The complete function of FhTLM during infection has yet to be explored but the on-going development of stable gene silencing techniques in *F*. *hepatica* will make this achievable [59]. A loss-of-function approach would be the best method to approach this subject, however there exists a number of hurdles, metacercariae have yet to be successfully treated with RNAi and as such NEJs treated with RNAi would need to be transplanted into the intestines of suitable hosts. The macrophage response to surgery has been shown to tend towards alternative activation, thus attempting to analyse the immune phenotype in such circumstances may prove difficult. A second complicating factor are the parasite-intrinsic effects of FhTLM [17], knock-out of FhTLM may yield a near-lethal or lethal phenotype, for reasons unrelated to host immunity, again complicating the analysis. A system for conditional gene targeting within the parasite metacercariae would best allow for natural infection and thus a faithful analysis of the resulting immune response; however these tools do not yet exist. Implementation of this technology will aid us in answering unresolved questions surrounding exact timing of FhTLM expression within the intestine of hosts, the full range of target cells and whether the effects of FhTLM are confined both physically and temporally confined to the intestine.

## Methods

### FhTLM and receptor fusion protein production

We have previously described the cloning and expression of FhTLM [17]. A pET28-based construct (Novagen) was used to express a 6XHis-Tagged protein in BL21 *E*. *coli* (Novagen) using kanamycin and chloramphenicol to select for transformed bacteria. Recombinant protein was purified using a Nickel resin column (Sigma-Aldrich). Recombinant proteins were subject to two rounds of phase separation prior to use [60]. To generate the receptor-fusion proteins the bovine TGFβRI extracellular domain sequence from nucleotide (nt) +88 to +331 and TGFβRII extracellular domain sequence from nt +139 to +453 relative to the translation initiation site (+1) were PCR amplified using specific primers. Using a forward primer with a NCOI restriction site incorporated and reverse primer with a Bg1II restriction site incorporated for TGFβRI and forward primer with an EcoRI restriction site incorporated and reverse primer with a Bg1II restriction site incorporated for generation of TGFβRII (See S1 Table). The amplified extracellular domains of TGFβRI was sub-cloned into the *NCOI* and *Bg1II* and TGFβRII ED into *EcoR1* and *Bg1II* multi cloning site of the pFUSE-hIgG1-Fc2 vector (InvivoGen, UK) respectively. Following ligation and confirmation of insertion plasmids were used to chemically transform *E*. *coli* DH5α cells. The transformed cells were grown on LB agar plates supplemented with 50μg/ml Zeocine (InvivoGen, UK) at 37°C overnight. Plasmid was purified and used to transfect mammalian HEK-293 cells (Invivogen UK) maintained in DMEM (Sigma Aldrich) supplemented with 10% FCS (Sigma Aldrich), 100 μg/ml penicillin, 100 μg/ml streptomycin and grown to 80% confluency. Transfection with recombinant plasmids was carried out using jetPRIME DNA and siRNA transfection reagents (Polyplus-transfection, USA) as per manufacturer’s instructions. The day before transfection cells were seeded into 6 well culture plate at 2x10^5^ cells /well, DMEM medium were added to final volume of 2ml per well and incubated at 37°C overnight 5% CO_2_. 2 μg of plasmid was diluted into 200 μl of jetPRIME buffer and 4 μl of jetPRIME were added to each well for transfection. The transfection medium were replaced with complete DMEM medium after 4 hrs and incubated at 37°C for 72 hrs. A positive control GFP reporter plasmid, Pc-gfp-c2 (Clontech), was used in parallel to confirm transformation. After 72 hrs the positive controls was assessed under an inverted microscope (LEICA DMIL). Positive cells were cloned under limiting dilution conditions, using zeocine (InvivoGen) as a selective.

### Direct TGF-β receptor-fusion and competition assays

96-well plates was coated with 50 μl of hTGF-β1 or FhTLM, concentration indicated on figures, at room temperature overnight. The plate was washed three times with 0.05% Tween/PBS. Additional protein binding site were blocked by adding 200 μl of 4% BSA-PBS and incubatedfor 1 hr at room temperature. TGFβ-RI and RII Fc fusion proteins were used at 1.25μg/mL and supernatant from non-transfected HEK were used as negative control and added to the wells of the plate and incubated for 1 hr at room temperature. HRP-conjugated Anti-human IgG1 Ab (HP6070, Life Technology) at a concentration of 5 μg/ml. Colour was developed with TMB substrate and the reaction was stopped with 1% HCL; absorbance was measured at 450nm using micro-plate reader (LT-4000, Labtech, UK).

To estimate the avidity of the FhTLM interaction with Fc fusion proteins of the bTGFβ-RIED and RIIED, potassium thiocyanate (KSCN) was introduced into the ELISA to disrupt the binding between the bovine receptors and the recombinant FhTLM protein. ELISA was performed as stated above using FhTLM as coating antigen at concentration of (500 ng/ml). After addition and incubation with (1.25 μg/ml) of Fc fusion proteins, different concentrations (1, 2, 3, 4, 5 and 0 M) of KSCN were added to each well and incubated for 1 hr at RT. Thereafter the binding and optical densities were measured as above. To determine the extent of competition between FhTLM and TGF-β in the context of binding to receptor fusion proteins, FhTLM or TGF-β were coated on plates at 400ng/mL. After this the opposite increasing concentrations of competing protein were incubated with the Receptor-Fc fusion in solution at 37°C for 1hr, then added to the plate and the ELISA proceeded as above.

### Luciferase Assay

Mink Lung epithelial cells (MLECs—a gift from Prof D Rifkin, New York University) were maintained in T25 flask (Sarstedt) containing 5 ml of Dulbecco’s modified Eagle’s Media (DMEM) (Sigma-Aldrich) supplemented with 10% of heat inactivated fetal calf serum (Sigma-Aldrich), penicillin (100 U/ml), streptomycin (100 U/ml), L-glutamine and 200 μg/ml, G418 (Sigma-Aldrich). For use in luciferase measurements cells were used at a concentration of 1.6x10^6^ cells/ml. The suspension was plated in 96 well tissue culture plates (Sarstedt) 100 μl/well. The culture plate was incubated at 37°C, 5% CO_2_ overnight for optimal cell attachment. Proteins including a TGF-β standard curve were added to cells in DMEM with 0.1% BSA. Luciferase was measured the luciferase Assay (Promega) on a BMG luminometer as per Abe et al [24].

### Macrophage Culture

Whole blood was collected under terminal exsanguination from healthy donor animals under a Home Office regulated schedule 1 procedure. CD14+ cells were isolated and cultured as before [61] IL-4 was used at 20ng/mL, LPS was used at 100μg/mL while FhTLM was either used at the indicated dose or 200ng/mL. Cells were stimulated for 6hr for RNA isolation or 48hrs for collection of supernatants and cell lysates.

RNAi knock-down *of tgfβRII* (NM_001159566.1) was conducted using the methods of Jensen et al [62] Briefly, siRNA oligos were designed by Sigma Aldrich and used at a final concentration of 100nM. Macrophages were cultured to maturity in 48 well plates at a density of 1 x 10^5^ cells and after 10 days were transfected with JetPrime media was replaced after 24hrs. Cells were tested for knock-down beginning at a further 24hrs after media change this using PCR primers designed against *tgfβRII;*Forward primer 5’ -GGACTATGAGCCTCCGTTCG- 3’ and reverse primer 5’–GGTTCCAGGAAGCATCGTCA- 3’. Alternatively, once an optimum time post-transfection was selected– 12hrs—stimulation was conducted.

### Fibroblast cultures and wound healing assay

The fibroblast cell line NIH 3T3 (A gift from Dr Janet Daly University of Nottingham) was routinely maintained and to conduct the scratch assay published methods were used. Briefly, 3x10^5^ cells were seeded into 6 well plates and incubated overnight. To scratch the monolayer, a linear scratch was made to the fibroblasts from the top of the well to the bottom using a 20μl pipette tip at time 0; plates were then incubated with the indicated proteins for 24hrs. Images of scratches were obtained using an inverted light microscope set to 5 x magnification. Lecia imaging software (leicra microsystems LTB Milton Keynes UK) was used to acquire digital images. ImageJ, was used to analyse the images (version 1.49v from National Institutes of Health, USA). Scratch areas were measured and compared by a blinded operator independent to the culture treatments for each well.

To conduct the CFU assay 6 cells/well were seeded in a 6-well plate, thereafter proteins were added and plates incubated for 10 days. Plates were stained with 0.5% crystal violet and imaged as above. CFUs were determined per well by an operator blinded to treatments before data analysis. In some experiments cells were co-cultured with SB-431542 a TGF-β RI kinase inhibitor (Tocris) and TGF-β or FhTLM with inhibitor at a final concentration of 5μM. Inhibitor stocks were prepared in DMSO and vehicle controls were prepared using an appropriate comparative dilution of DMSO.

### Immunofluorescence

For immunofluorescence staining cells were isolated and stimulated as above but grown on coverslips (Corning). Following stimulation plates were centrifuged at 300xg for 10 min following incubation, to collect cells on the cover slips. Medium were removed and cells were washed three times with 1XPBS. PBMCs were then fixed by in 4% paraformaldehyde for 15 min at room temperature and washed with 1XPBS. Afterward, cells were permeabilized for 10 min at room temperature with 0.5% Triton X-100 in PBS and washed with PBS. Cells were incubated for 1 hr at room temperature with a 1:100 dilution of primary polyconal rabbit anti-GATA1 (Santa Cruz sc-13053) or goat anti-pSmad2/3 (Santa Cruz sc-11769) or mouse anti-mannose receptor (ThermoFisher 2G11). After extensive washing with PBS, the cells were incubated for 1 h at room temperature in the dark with 1:1000 dilution of secondary anti-IgG-FITC. Slides were then washed with PBS, mounted with Vectashield (Vector Labs Ltd., UK) containing DAPI staining reagent. Images were captured using Leica DM500B microscope and DFC 350FX camera (Leica Microsystem Ltd., UK) using X40 and X63 magnification.

### Cytokine analysis, enzyme assays, qPCR

ELISAs or paired antibodies were used to detect cytokines were conducted as per manufacturer’s instructions the kits were as follows; IL-10 (CSB-E12917B) was purchased from Cusabio; IFN-γ (ESS0026B) from ThermoScientific; and IL-12 paired antibodies (CC301 –capture and CC326 –detection) were from AbD Serotec. Nitric oxide was measured using a Griess Reagent Kit (Promega) and arginase levels were determined using the method of [63]. To measure PD-L1 via qPCR RNA was isolated from cells 6hrs after stimulation and converted to cDNA using the GoScript Reverse Transcription Kit (Promega). Primers and conditions used are as previously reported [64].

### *F*. *hepatica* metacercariae excystment and ADCC killing

F. hepatica metacercariare were obtained from the University of Liverpool clonal strain FhepLiv and NEJs excysted as previously described [17]. Parasites were rested for 4hrs prior to use in the ADCC assay which was conducted as previously described by Piedrafita et al [57] and Van Milligen et al [65]. Briefly, macrophages were cultured as described above and mixed with rested NEJs in wells of a 48 well plate containing 40 NEJs and 2 x 10^5^ macrophages per well. Serum was collected from three cattle prior to infection and 13 weeks post infection, with 250 metacercariae of *F*. hepatica (Kind gift from Dr Divya Sachdev University of Nottingham). Sera was added to wells at a final concentration of 15% in a total volume of 250μl/well. Plates were incubated for 48hrs and NEJs were observed for viability by monitoring motility, absence of defined intestinal structures and exclusion of trypan blue. Parasites were only classified as non-viable if motility and intestinal structure were absent/not visible and trypan blue was taken up in the tegument. NO was measured in the same supernatant using the methods above. In some experiments, macrophages were pre-incubated with the inhibitor SB-431542 prior to FhTLM or TGF-β stimulation as described above.

### Statistical analysis

Data was entered into Prism 6.01 (Graphpad) for statistical analysis. Data was analysed using a 1-way Anova with post-test comparison using a Tukey correction. Apart from data in Fig 6 which was analysed using 2-way anova to determine the effect of serum type and macrophage stimulation. P values <0.05 were taken as significant and individual P values are listed in figure legends.

## Supporting Information

S1 Fig TGF-β luciferase assay on LFH. MELC luciferase reporter cells were used to test for the presence of TGF-like molecules in LFH. Cells were cultured in the presence of the indicated doses of LFH overnight, before luciferase was determined using a luciferase assay on a BMG luminometer. LFH was tested in triplicate cultures and at least 5 batches were tested.
(TIF)

S2 Fig Dose dependent induction of IL-10 in bovine macrophages. Bovine macrophages, 10^5^/well, were cultured in the presence of increasing doses of FhTLM as indicated. After 48hrs supernatants were collected and tested for IL-10 by ELISA. Results displayed here represent the mean +/- SD of triplicate cultures from a single donor, this experiment was repeated five times with similar outcomes.
(TIF)

S1 Table Forward and reverse primers used to amplify the extracellular portion of the corresponding bovine TGF-β RI or RII. Underlined text corresponds to the restriction site used for cloning.
(DOCX)
