# Supplementary figures and images for: A Trematode Parasite Derived Growth Factor Binds and Exerts Influences on Host Immune Functions via Host Cytokine Receptor Complexes
Source: PLoS Pathog. 2016 Nov 2;12(11):e1005991. doi: 10.1371/journal.ppat.1005991 (PMC5091765; doi:10.1371/journal.ppat.1005991)

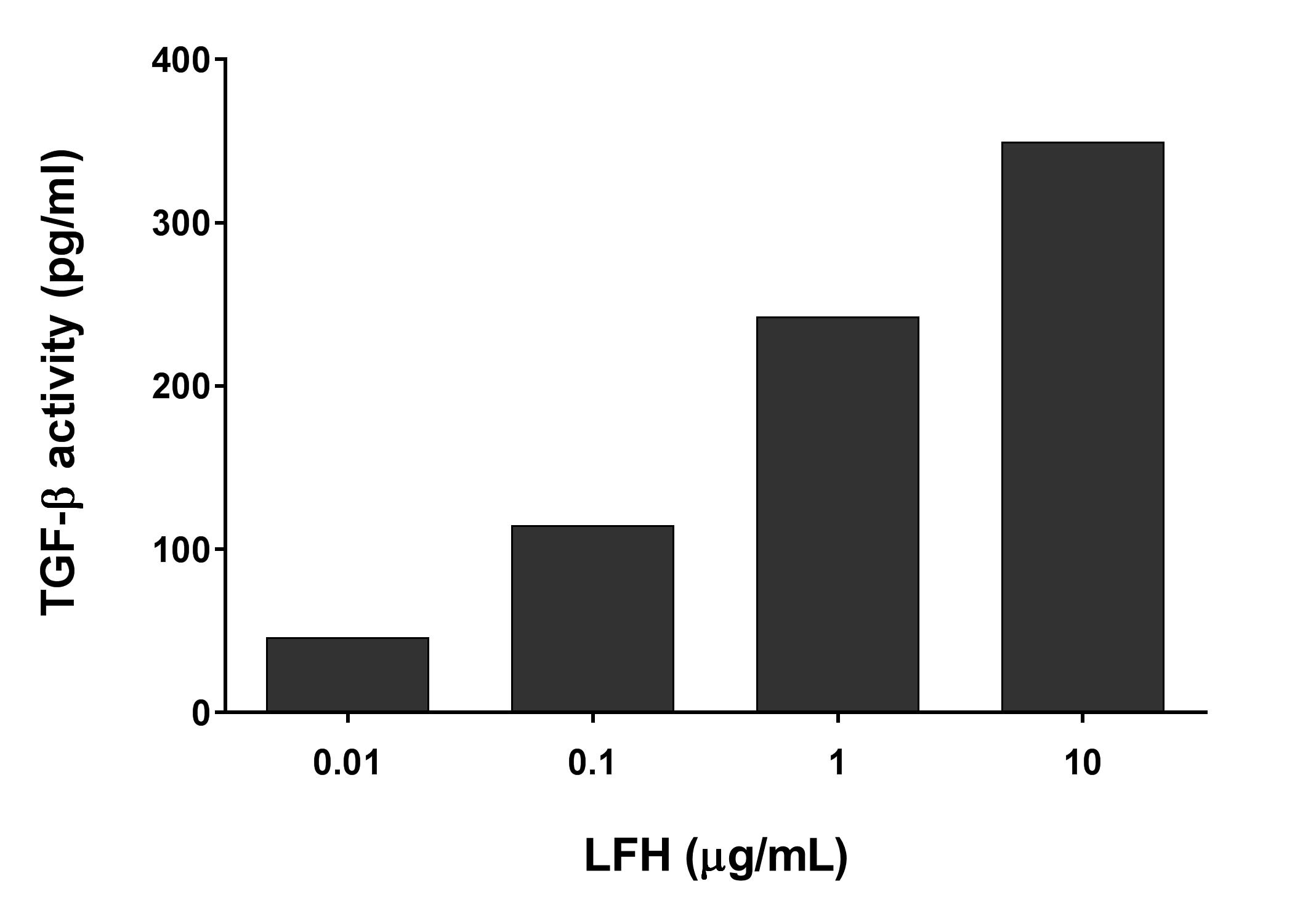

Supplement: S1 Fig — MELC luciferase reporter cells were used to test for the presence of TGF-like molecules in LFH. Cells were cultured in the presence of the indicated doses of LFH overnight, before luciferase was determined using a luciferase assay on a BMG luminometer. LFH was tested in triplicate cultures and at least 5 batches were tested. (TIF) [file ppat.1005991.s001.tif]

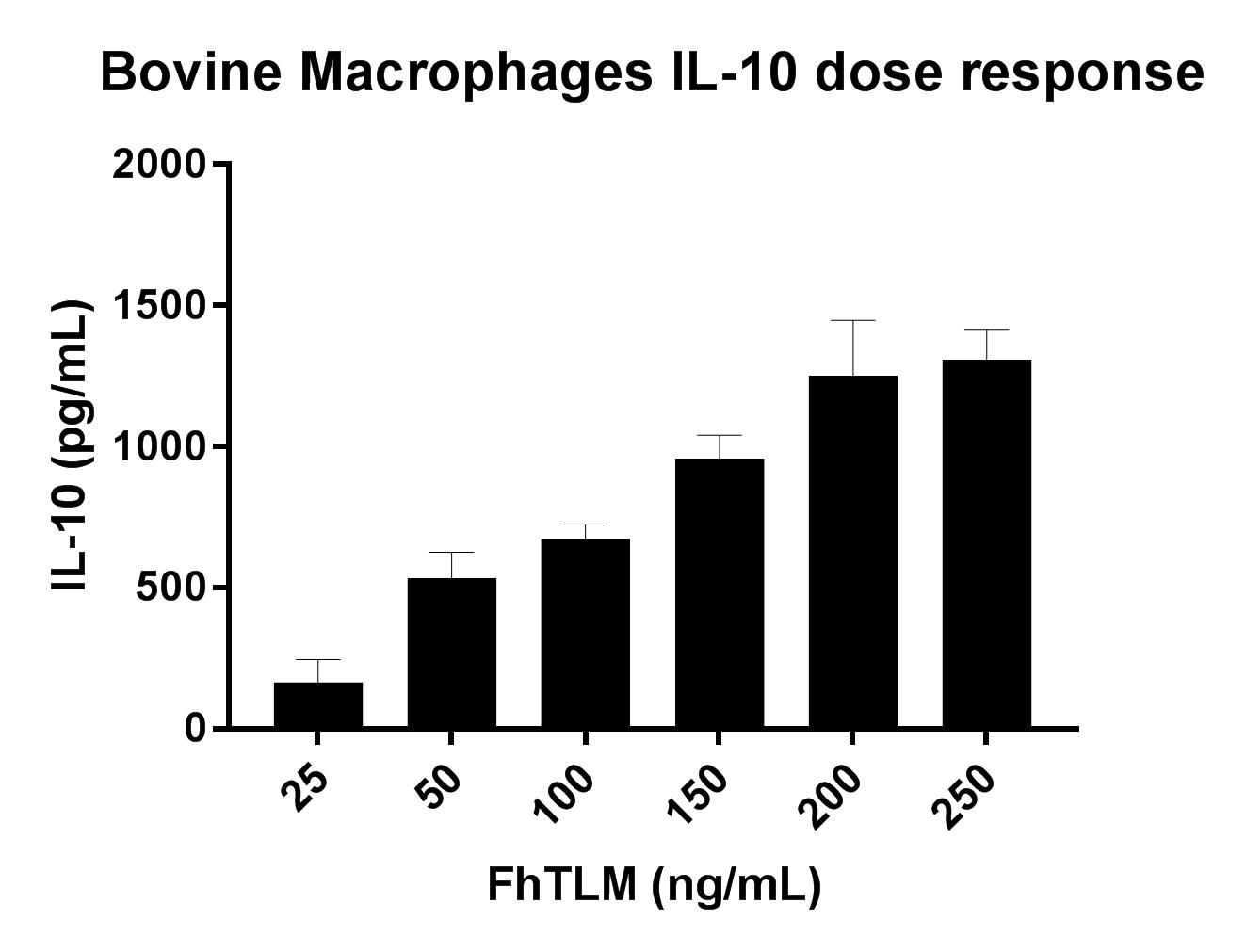

Supplement: S2 Fig — Bovine macrophages, 105/well, were cultured in the presence of increasing doses of FhTLM as indicated. After 48hrs supernatants were collected and tested for IL-10 by ELISA. Results displayed here represent the mean +/- SD of triplicate cultures from a single donor, this experiment was repeated five times with similar outcomes. (TIF) [file ppat.1005991.s002.tif]
