# Supplementary material for: A Trematode Parasite Derived Growth Factor Binds and Exerts Influences on Host Immune Functions via Host Cytokine Receptor Complexes
Source: PLoS Pathog. 2016 Nov 2;12(11):e1005991. doi: 10.1371/journal.ppat.1005991 (PMC5091765; doi:10.1371/journal.ppat.1005991)
Supplement: S1 Table — Underlined text corresponds to the restriction site used for cloning. (DOCX) [file ppat.1005991.s003.docx]

# **Supporting Table 1:** Primers used in receptor-fc fusion cloning.

| **Target** | **Sequence (5’ – 3’)** |
| --- | --- |
| Forward TGFβ-R1 *NCOI* | TGGCCATGGCAGTGTTTTTGCCACCTTG |
| Reverse TGFβ -R1 *Bg1II* | GCCGATCTTGATGGCTTTCCAACAGTTG |
| Forward TGFβ-R2 *EcoR1* | ATTGAATTCGCTGTCGCAGCTGTGTAAG |
| Reverse TGFβ-R2 *Bg1II* | ACCAGATCTTTACTGCTGGTGTACTCTTC |

Underlined text corresponds to the restriction site used for cloning.
